# Improved *In vivo* Assessment of Pulmonary Fibrosis in Mice using X-Ray Dark-Field Radiography

**DOI:** 10.1038/srep17492

**Published:** 2015-12-01

**Authors:** Andre Yaroshenko, Katharina Hellbach, Ali Önder Yildirim, Thomas M. Conlon, Isis Enlil Fernandez, Martin Bech, Astrid Velroyen, Felix G. Meinel, Sigrid Auweter, Maximilian Reiser, Oliver Eickelberg, Franz Pfeiffer

**Affiliations:** 1 Lehrstuhl für Biomedizinische Physik, Physik-Department & Institut für Medizintechnik, Technische Universität München, Garching, Germany; 2 Institute for Clinical Radiology, Ludwig-Maximilians-University Hospital Munich, Munich; 3 Comprehensive Pneumology Center, Institute of Lung Biology and Disease, Member of the German Center for Lung Research (DZL), Helmholtz Zentrum München, Neuherberg, Germany; 4 Institute for Experimental Pneumology, Ludwig-Maximilians-University Hospital Munich, Munich; 5 Department of Medical Radiation Physics, Lund University, Lund, Sweden

## Abstract

Idiopathic pulmonary fibrosis (IPF) is a chronic and progressive lung disease with a median life expectancy of 4–5 years after initial diagnosis. Early diagnosis and accurate monitoring of IPF are limited by a lack of sensitive imaging techniques that are able to visualize early fibrotic changes at the epithelial-mesenchymal interface. Here, we report a new x-ray imaging approach that directly visualizes the air-tissue interfaces in mice *in vivo.* This imaging method is based on the detection of small-angle x-ray scattering that occurs at the air-tissue interfaces in the lung. Small-angle scattering is detected with a Talbot-Lau interferometer, which provides the so-called x-ray dark-field signal. Using this imaging modality, we demonstrate-for the first time-the quantification of early pathogenic changes and their correlation with histological changes, as assessed by stereological morphometry. The presented radiography method is significantly more sensitive in detecting morphological changes compared with conventional x-ray imaging, and exhibits a significantly lower radiation dose than conventional x-ray CT. As a result of the improved imaging sensitivity, this new imaging modality could be used in future to reduce the number of animals required for pulmonary research studies.

Pulmonary fibrosis may develop in response to a variety of causes, e.g. in the frame of collagen/vascular diseases, in response to inorganic (asbestos, silicosis) or organic (allergens) dusts, or following medical interventions, such as chemo- or radiotherapy. In contrast, the cause for pulmonary fibrosis in idiopathic pulmonary fibrosis (IPF) remains unknown up-to-date. IPF, which alone may affect up to 150,000 patients in the European Community, is characterized by an average survival time of 4–5 years from diagnosis and represents the most aggressive form of lung fibrosis^1^,^2^,^3^,^4^.

The main cause of death in patients with IPF is respiratory failure. Although one treatment has recently been approved in Europe for IPF, reversal of lung fibrosis is currently impossible, and lung transplantation remains the only therapeutic intervention in end-stage IPF. This emphasizes a dire need for new technologies for the early detection and accurate monitoring of IPF. In addition, the development of new therapeutic strategies demands sensitive methods suitable for longitudinal monitoring and quantification of disease progression. Currently, the gold standard for assessing the structure of the lung, histology, only offers endpoint measurements at the time of biopsy or transplantation. While pulmonary function tests are routinely used to monitor disease progression, they are not suitable for assessing short-term changes in the breathing parameters and lag behind histological changes^5^.

To address this issue, different biomedical imaging techniques like conventional x-ray CT^6^,^7^,^8^, PET/SPECT-CT^9^,^10^,^11^,^12^ and MRI^13^,^14^,^15^,^16^ have been described for the monitoring of fibrosis (especially in small animals), but face considerable limitations. Both CT and MRI cannot resolve individual alveoli and the assessment of the disease severity is based solely on the detection of larger areas of scarred tissue. As such, current imaging modalities exhibit low sensitivity and progression predictability, especially at an early stage of the disorder. Apart from this significant drawback, x-ray CT (and particularly high-resolution CT), along with PET/SPECT-CT, are not suitable for regular monitoring purposes due to high patient radiation exposures. MRI, on the other hand, yields only low spatial resolution and is limited by long scanning times, high operation costs, and low regional availability.

In the present study, we report on a new approach for *in vivo* quantitative assessment of air-tissue interfaces via x-ray dark-field imaging. To acquire an x-ray dark-field image, a three-grating Talbot-Lau interferometer is introduced into the x-ray beam^17^,^18^,^19^. This imaging approach makes it possible to obtain x-ray phase and scattering information without the use of highly coherent synchrotron sources. A number of images are acquired while moving one of the gratings perpendicular to the beam propagation direction over one grating period^20^. Analysis of the image sequence acquired with and without the sample in the beam yields three distinct images: conventional x-ray transmission, differential phase contrast, and dark-field scatter contrast. While x-ray transmission reveals information about the absorbing properties of the material, dark-field imaging quantifies the small-angle scattering in the sample^21^,^22^.

Due to a strong difference in the refraction index between air and lung tissue, the x-rays are refracted on each air-tissue interface. Numerous air-tissue interfaces in a healthy lung lead to a strong dark-field signal, as observed in healthy *in vivo* mice^23^,^24^. A number of studies have been recently performed in order to evaluate if x-ray refraction and scattering information could be used for an improved diagnosis of pulmonary disorders. Early studies, conducted at synchrotrons have revealed that the x-ray phase and scattering information can indeed be used for a superior evaluation of lung tissue^25^ and e.g. improved diagnosis of pulmonary pneumonitis^26^ or emphysema in *ex vivo* samples^27^. Other studies have used the enhanced contrast for visualization of airways in mice affected by different pulmonary disorders^28^. Similar results as obtained at the large-scale monochromatic source for excised *ex vivo* murine emphysema lungs have been also recently achieved using a polychromatic laboratory x-ray source^29^. Finally, it was reported that in spite of overlaying structures and *in vivo* breathing motion, x-ray dark field provides a significantly better visulaization of different stages of emphysema than conventional tranmission imaging^30^,^31^.

Unlike emphysema, the possibility to visualize morphological changes associated with pulmonary fibrosis using x-ray dark-field imaging has not been studied so far. Therefore, the goal of the present study was to investigate the potential of x-ray dark-field radiography for visualization of morphological lung changes associated with pulmonary fibrosis in small animals.

## Results

The feasibility of diagnosing pulmonary fibrosis based on x-ray dark-field radiographs was demonstrated using ten control animals and ten mice with pulmonary fibrosis, induced by orotracheal instillation with 2.5 units/kg body weight of bleomycin, as described earlier^32^. All mice were imaged 21 days after instillation and pulmonary function tests were performed after radiography. Shortly thereafter the animals were sacrificed for histological analysis.

**Figure 1:**
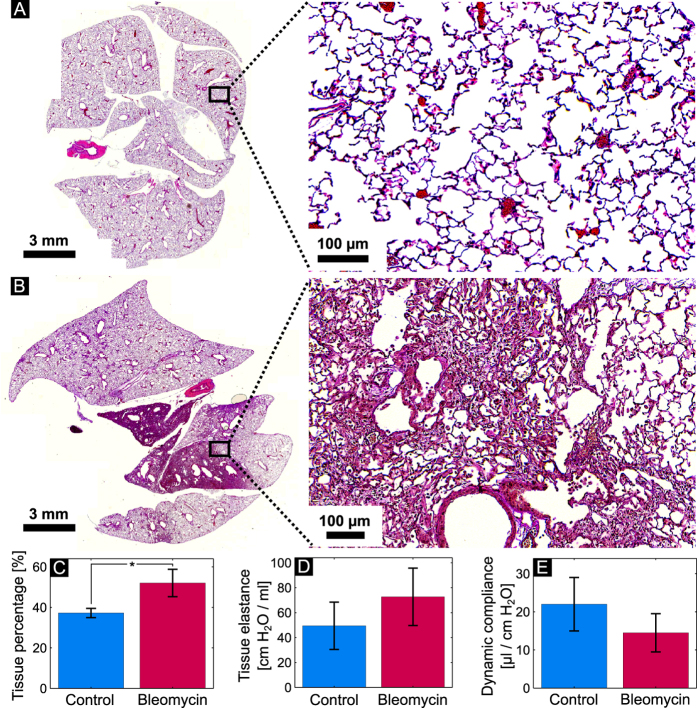
Histological slice (and a magnified section) of a control lung (A) and a lung treated with bleomycin (B). The quantified lung tissue percentage for all the animals in the control and the bleomycin groups is presented in (**C**). (**D**,**E**) reveal the tissue elastance and dynamic compliance for the two animal groups obtained from the pulmonary function tests.

Histopathology of the harvested lungs revealed a clear difference between the control and the bleomycin group. Figure 1 shows a typical histological slice (and a magnified section) of a healthy lung (A) and a lung showing fibrotic changes (B). The control lung is clearly constituted by a healthy, dense alveolar network. By contrast, in case of the animal treated with bleomycin the distal airspaces are filled with extracellular matrix components, with severe consequences for the functionality of the lung. Thus, typical morphological changes associated with pulmonary fibrosis were observed on histological slices, so that it can be concluded that any changes observed on the x-ray images are associated with the presence of fibrosis.

The morphological changes caused by bleomycin were quantified from the histological slices by determining the tissue percentage as described in the materials and methods section. The obtained mean values, along with the standard deviations for the two groups are shown in Fig. 1C. Histological findings yield a significant difference (p < 0.01) between the two groups. A higher standard deviation of the tissue percentage for the fibrotic animals highlights the varying severity of the induced pulmonary disorder. The lung function tests, on the other hand, failed to provide a significant difference between the control and the bleomycin animal groups. A trend, but no significant difference, could be observed for both tissue elastance (p = 0.05) (Fig. 1D) and dynamic compliance (Fig. 1E) (p = 0.05). The high standard deviation in the control group highlights the strong variability of spirometry and emphasizes the need for a more sensitive diagnostic tool for the detection of early fibrotic changes in the lung.

**Figure 2:**
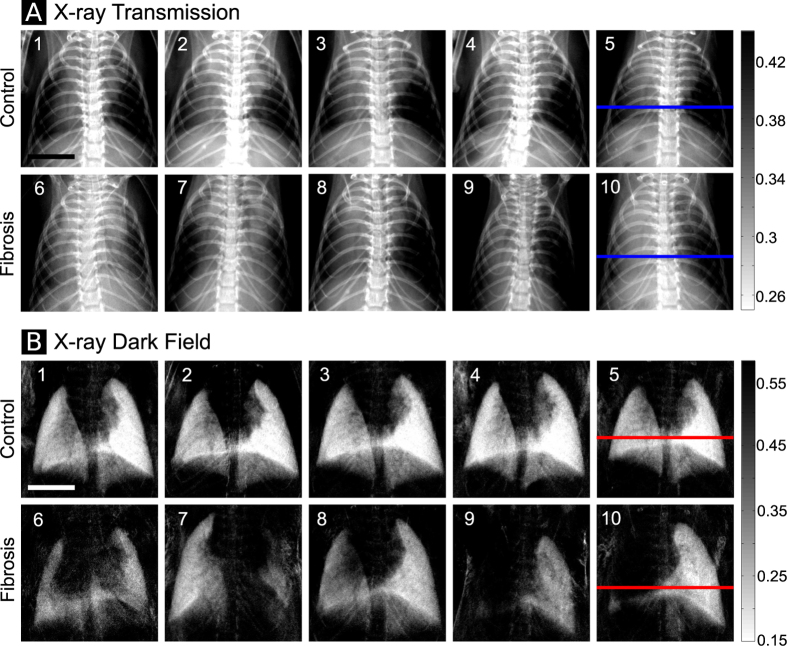
(**A**) Conventional x-ray transmission radiograms of five control mice (animals 1–5, top row) and five mice with pulmonary fibrosis (animals 6–10, bottom row). (**B**) X-ray dark-field radiograms of the same animals. The scale bar corresponds to 5 mm.

X-ray transmission and dark-field radiograms calculated from the identical image sequences acquired for five control and fibrotic mice are shown in Fig. 2.

**Figure 3:**
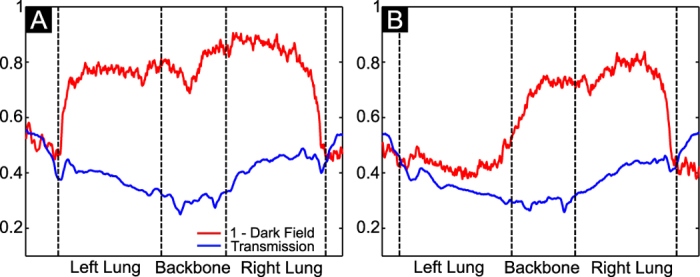
Transmission (blue) and dark-field (red) line profiles for a healthy (A) and fibrotic mouse (B). The position of the analyzed line is shown on the chosen radiographies in Fig. 2, mice 5 and 10.

**Figure 4:**
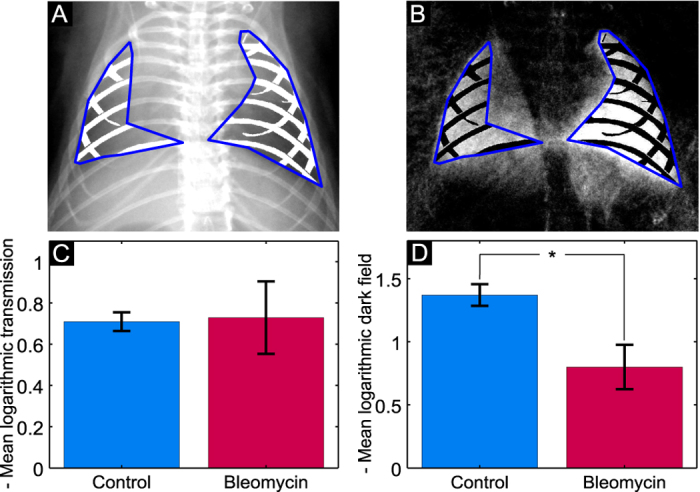
Representative masks used for the signal quantification on the transmission (A) and dark-field (B) image. The area inside the blue line without the ribcage was taken into account. (**C,D**) show the negative mean logarithmic transmission and dark-field signals for the control and the bleomycin group, respectively.

It can be easily seen that the mice treated with bleomycin developed pulmonary fibrosis of varying severity. We observe that severe pulmonary fibrosis is clearly visible on the conventional x-ray transmission images, as well as the dark-field radiograms (e.g. mouse 9). In case of severe fibrosis, alveolar airspaces and perialveolar regions are subject to significantly increased extracellular matrix (ECM) and coagulation product deposition (as shown in Fig. 1B). Therefore, severely affected regions of the lung can be identified as regions of decreased x-ray transmission. The dark-field signal of the lung originates from the x-ray refractions on multiple air-tissue interfaces^23^. Both simulations^33^ and experimental results^27^,^29^ have shown that the reduction of the number of interfaces results in decrease of x-ray scattering. Therefore, control lungs appear bright on the dark-field images (Fig. 2B, mice 1–5) and regions of the lung affected by fibrosis yield significantly less scattering and negligible dark-field signal. In cases of mild and moderate fibrosis (e.g. Fig. 2, animals 6 and 8), x-ray transmission images do not provide a clear signal difference between healthy and fibrotic lung tissue. By contrast, alterations in the lung parenchyma are directly and more sensitively visualized on the dark-field radiograms. Thus, changes in the lung parenchyma can be precisely observed for mouse 6 & 8 in the dark-field but not on the transmission images. To further highlight the superiority of x-ray dark field for the detection of fibrotic changes, a line plot through a healthy (Fig. 2 mouse 5) and a fibrotic (mouse 10) animal for both signals is shown in Fig. 3. On this plot both signals are scaled between 0 and 1. It can be observed that the difference in signal between the right healthy lung and fibrotic left lung in Fig. 3B is very prominent in the dark-field but not in the transmission signal. To estimate the improvement in diagnostic value of x-ray dark-field imaging over conventional transmission, the diagnostic power of both imaging modalities was quantified for the control and the fibrotic animals. For the analysis a mask was manually created on the radiograms including all of the lung tissue, excluding the heart shadow and the ribcage. Figure 4A,B show a typical identical mask for a transmission and a dark-field radiogram.

Using these masks, the x-ray transmission and dark field were quantified for all the animals and the mean transmission and dark field group values are presented in Fig. 4C,D, respectively. It can be easily appreciated that the transmission signal does not provide sufficient discrimination between the control and the bleomycin group (p = 0.43). The quantified dark field provides a significant difference (p < 0.01) and confirms the qualitative impression. It is also noted that the overlying structures such as fat, skin, etc. contribute significantly to the x-ray transmission signal and complicate an accurate diagnosis. This is the reason for the low discriminative power of the transmission signal. These overlying structures are, by contrast, almost invisible in the dark field.

The presented results prove that it is possible to detect pulmonary fibrosis in mice using x-ray dark-field imaging. The results further suggest that x-ray dark-field imaging is well-suited for sensitive and accurate diagnosis of pulmonary fibrosis, even with only mild and moderate pathological differences.

**Figure 5:**
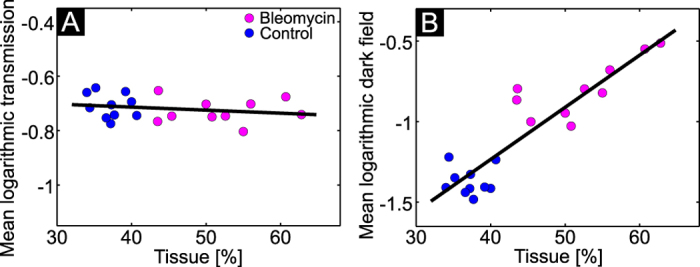
Correlation between the quantified tissue percentage and the mean logarithmic x-ray transmission (A) and the dark-field signal (B). The black line is a linear fit to the data.

In order to evaluate how both transmission and dark-field signals scale with the progression of fibrosis, a scatterplot was created correlating both imaging signals with the tissue percentage, obtained by terms of histological analysis. Similarly to x-ray transmission, the dark-field signal scales with a negative exponential with the sample thickness^21^,^22^. Hence, the logarithm of both signals was considered for the analysis. The resulting scatterplots are shown in Fig. 5. Control and fibrotic animals are marked with blue and magenta color, respectively. A linear fit was added to the plots for a better visualization of the linear correlation, predicted by simulations^33^.

As histology revealed, healthy mice have a lung tissue percentage of around 35% and this percentage increases with increasing severity of fibrosis. The transmission signal gradually decreases with the severity of fibrosis, however, it shows only a low correlation with histological changes (Fig. 5A). Pearson’s and Spearman’s correlation coefficients for tissue percentage and transmission signal are only −0.16 and −0.22 respectively. On the other hand, the signal change in the dark field is much larger. There is a clear linear correlation between the dark-field signal and tissue percentage. Pearson’s and Spearman’s correlation coefficients are 0.91 and 0.83, respectively. The presented observation suggests that bleomycin-induced pathological changes can be monitored more sensitively with dark-field imaging compared to conventional transmission imaging.

## Discussion

In this study, we have demonstrated for the first time the feasibility to diagnose end-stage pulmonary fibrosis using x-ray dark-field radiography. It has been shown that the dark-field modality is significantly more sensitive to fibrotic morphological changes in the lung than conventional x-ray imaging, and hence could enable earlier diagnosis of the disorder. Contrary to other imaging modalities, x-ray dark-field radiography yields direct access to the information about the state of air-tissue interfaces in the lung. It is also very likely – but not yet proven here directly—that the signal scales well with gaseous exchange measures, since the larger the number or area of tissue-air interfaces, the better the oxygen exchange. This information has not been accessible so far with conventional x-ray and MRI imaging. Therefore, x-ray dark-field radiography has the potential to facilitate new insight into the pathology and development of pulmonary fibrosis. The total dose necessary for the acquisition of dark-field and transmission radiograms in our study is estimated with 1.4 mGy. Therefore, it can be assumed that x-ray dark-field radiography can be used for longitudinal small animal studies without further modifications to the imaging system. The high-resolution micro CT used so far for the assessment of pulmonary fibrosis in mice, by contrast, requires around 850 mGy^8^ and is not suitable for longitudinal studies.

The not significant discrimination between control and fibrotic groups in the lungs function test agrees with the results observed in humans, where pulmonary function tests have been reported to show only moderate correlation with the severity of the disorder^5^,^34^,^35^. In case of small-animal experiments the results of the lung function tests further strongly depend on the depth of the anesthesia and the dead volume introduced by the tracheostomy. Consequently, a high variability of the results of pulmonary function tests can be expected and it is not straightforward to diagnose pulmonary fibrosis in mice based on spirometry alone.

It has been further demonstrated that x-ray dark-field signal can be used as a quantitative figure of merit for changes in the lung parenchyma. This opens new horizons for quantitative therapy studies. The low correlation of the transmission signal with tissue percentage in Fig. 5A can be explained by the strong influence of the overlaying structures (like skin, fat layer, etc.) on the transmission signal. The dark-field signal is compromised only by the animal’s fur, which gives quite a homogeneous background. Thus, in the future, dark-field radiography should be applied to longitudinally monitor small animal therapy studies, with special focus on visualization of areas with recovering lung functionality. By quantitatively determining the individual baseline at the time point of therapy application, it should be possible to significantly reduce the number of animals required for the experiments (compared to previous studies that used less-sensitive tools) and to individually track morphological changes.

In earlier studies, pulmonary emphysema has been shown also to cause a similar decrease in small-angle x-ray scattering to pulmonary fibrosis^29^,^30^. Consequently, further research should focus on considering not only sensitivity of x-ray dark-field, but also its specificity. In particular studies could identify if the combination of x-ray transmission and dark field provides a sufficient footmark to discriminate between emphysematous and fibrotic tissue.

At the moment, dark-field imaging is limited to small-animal studies only. Therefore, further technical developments are required to transfer this imaging modality to a larger field-of-view and to higher energies, as required for human applications. The development of dark-field imaging for human studies would be a significant achievement, as the superior sensitivity and significantly less radiation exposure of dark-field radiography compared to CT would enable early screening approaches in individuals with a higher risk to develop IPF, such as familial cases.

## Materials and Methods

### Small animal protocol

Animal experiments were performed with permission of the Institutional Animal Care and Use Committee of the Helmholtz Zentrum Munich and carried out in accordance with national (Gesellschaft für Versuchstierkunde—Society for Laboratory Animal Science) and international (Federation for Laboratory Animal Science Associations) animal welfare guidelines. The Institutional Animal Care and Use Committee of the Helmholtz Zentrum Munich approved all the experimental protocols. For the study pathogen-free female C57BL/6N mice (Charles River, Sulzfeld, Germany) aged six- to eight-weeks were used. To induce fibrosis, ten mice were orotracheally instilled with 2.5 units/kg body weight of bleomycin dissolved in 80 μl phosphate-buffered saline (PBS). Ten control animals received 80 μl sterile PBS. The animals were imaged 21 days after instillation and sacrificed shortly thereafter for histological analysis.

Mice were anesthetized using intraperitoneal injection of medetomidine (500 μg/kg), midazolam (5 mg/kg), and fentanyl (50 μg/kg) for the bleomycin application, imaging and pulmonary function tests. Before sacrificing the animals a flexiVent system (Scireq Inc, EMKA Technologies, Paris, France) was used to acquire *in vivo* pulmonary function test data including tissue elastance and dynamic compliance.

### X-ray transmission and dark-field imaging

A prototype small-animal scanner^36^,^37^, developed in collaboration with Bruker microCT (Kontich, Belgium), was used for small-animal x-ray transmission and dark-field imaging. The rotating gantry built into the scanner consists of an x-ray source (RTW, MCBM 65B-50 W, focal spot size approximately 50 μm in diameter), a flat-panel detector (Hamamatsu, C9312SK-06, GOS scintillator, with 50 μm pixel size) and a three-grating Talbot-Lau interferometer. During imaging the animal’s body temperature and breath rate was monitored and a built-in warm-air fan was used to prevent the animals from cooling down. For image acquisition the x-ray source was operated at 35 kVp (source power 17 W). Five different images were acquired while stepping the source grating^18^ over one grating period. The obtained images were subsequently processed using an in-house written Matlab code to obtain the conventional x-ray transmission as well as dark-field images. The image processing was based on Fourier signal decomposition^20^. The exposure time for each image was five seconds. The spatial resolution of the scanner is 60 μm (10% MTF). During imaging mice were breathing freely.

### Histology

Lungs were fixed in a 4% of paraformaldehyde, later decalcified in a 10% Ethylenediaminetetraacetic acid (EDTA) solution for 5 days. Dehydrated samples were subsequently embedded in paraffin. Approximately fifteen 10 μm slices were prepared from each lung. Finally, the slices were stained using the Elastica-van-Gieson (EVG) protocol. Prepared slices were then scanned using an Olympus BX51 (Olympus, Hamburg, Germany) microscope to create digital images. The severity of the fibrosis was evaluated based on the tissue percentage on the histological slices. For tissue percentage quantification the light microscope was equipped with a computer-assisted stereologic toolbox (newCAST; Visiopharm, Hørsholm, Denmark). Histological sections were magnified 200-fold and overlaid with a line grid. Points hitting lung parenchymal tissue (P_tissue_) or lung parenchymal air (P_air_) were counted for 50 fields of view per lung. The lung tissue percentage was calculated according to equation % Tissue = ∑P_tissue _× 100/∑P_tissue_ + ∑P_air_.

### Data analysis

The parameters between the control and bleomycin groups were compared using the Mann-Whitney test.

## Additional Information

**How to cite this article**: Yaroshenko, A. *et al.* Improved *In vivo* Assessment of Pulmonary Fibrosis in Mice using X-Ray Dark-Field Radiography. *Sci. Rep.* **5**, 17492; doi: 10.1038/srep17492 (2015).
